# The effects of dexmedetomidine for patient-controlled analgesia on postoperative sleep quality and gastrointestinal motility function after surgery: A prospective, randomized, double-blind, and controlled trial

**DOI:** 10.3389/fphar.2022.990358

**Published:** 2022-10-10

**Authors:** Xin Sui, Yue Wang, Mingxin Jin, Kun Li, Ge Jiang, Ailing Song, Zhaoyi He, Chengke Yin, Jingshun Zhao, Liping Wang, Fei Han

**Affiliations:** ^1^ Department of Anesthesiology, Harbin Medical University Cancer Hospital, Harbin, Heilongjiang, China; ^2^ Department of Pain Medicine, Shanghai Fourth People’s Hospital Affiliated to Tongji University School of Medicine, Shanghai, China; ^3^ Department of Anesthesiology, Cancer Hospital Chinese Academy of Medical Science, Shenzhen Center, Shenzhen, China

**Keywords:** dexmedetomidine, patient-controlled analgesia, postoperative sleep quality, postoperative gastrointestinal motility function, colorectal cancer surgery

## Abstract

**Background:** Postoperative poor sleep quality and decreased gastrointestinal motility function are common clinical problems. This study investigated the effects of dexmedetomidine (DEX) combined with sufentanil for patient-controlled analgesia (PCA) on postoperative sleep quality and gastrointestinal motility function after surgery in patients with colorectal cancer.

**Methods:** Patients undergoing colorectal cancer surgery were randomly divided into three groups, DEX 0, 200, or 400 μg, each combined with sufentanil 150 μg for PCA immediately after surgery. The primary outcome was sleep quality in the first 7 days after surgery based on the Athens Insomnia Scale (AIS) score. The secondary outcome was postoperative gastrointestinal motility recovery evaluated by the time of first flatus, first feces and first diet. Postoperative pain intensity, side effects and the length of postoperative hospital stay were also compared among groups. The study was registered with the Chinese Clinical Trial Registry (https://www.chictr.org.cn/enIndex.aspx, ChiCTR2000032601).

**Results:** Ultimately, 210 cases were included. Sleep quality was better in the DEX 200 μg group and DEX 400 μg group than in the DEX 0 μg group. Overall, in the DEX 200 μg group and DEX 400 μg group, the AIS score (*p* < 0.05) and the incidence of sleep disturbance (7.3%, 4.5% vs. 19.6%, *p* < 0.001) were lower than those in the DEX 0 μg group in the first 7 days after surgery. There were no significant differences in postoperative gastrointestinal motility among the three groups in the total surgical categories (*p* > 0.05). In the laparoscopic surgery patients of each group, the time of postoperative first flatus (*p* = 0.02) and first feces (*p* = 0.01) was significantly longer in the DEX 400 μg group than in the DEX 0 μg group. There were no differences in postoperative pain intensity, side effects or length of postoperative hospital stay (*p* > 0.05).

**Conclusion:** The continuous infusion of DEX (200 or 400 μg) for PCA significantly improved postoperative sleep quality after colorectal cancer surgery. DEX (200 μg) was better at improving postoperative sleep quality without affecting gastrointestinal motility function than DEX (400 μg) in patients who underwent laparoscopic colorectal cancer surgery.

**Clinical Trial Registration:** https://www.chictr.org.cn/enIndex.aspx, identifier ChiCTR2000032601

## Introduction

Sleep is essential for energy conservation, temperature regulation, immune response and brain recovery. Postoperative sleep disturbance is a common clinical problem in surgical patients and is related to surgery, anesthesia and environment (Jiang et al., 2018). Poor sleep quality increases the incidence of postoperative delirium and postoperative cognitive dysfunction, increases the incidence of cardiovascular events and delays the postoperative recovery process (Aldecoa et al., 2017; Huang et al., 2021). Sleep disturbance is the second most bothersome symptom in cancer patients, and it increases the chances of cancer recurrence (Otte et al., 2015). Many attempts have been made to alleviate severe sleep disturbance after surgery by eliminating noise and light through the use of eye masks or earplugs in surgical wards, but the effectiveness of these strategies is limited, and adjuvant medication is needed in some circumstances (Ouslander et al., 2006; Leong et al., 2021). Pharmacological interventions such as benzodiazepines, propofol or analgesics, are used to improve postoperative sleep quality. Unfortunately, these drugs may produce sleep architecture disruption and increase the incidence of postoperative delirium (Pandharipande and Ely, 2006).

Dexmedetomidine (DEX) is a potent and highly selective α_2_ adrenergic agonist with sedative, analgesic and sympatholytic properties (Lee, 2019). In contrast to other commonly used sedative and anesthetic agents to induce neuronal apoptosis, DEX has shown neuroprotective effects (Duan et al., 2014). The sedation induced by the administration of DEX neurophysiologically approximates natural sleep (Akeju et al., 2018). Some clinical trials have demonstrated that the intraoperative use of DEX can decrease the incidence of sleep disturbance in postoperative patients (Wu et al., 2016; Chen et al., 2017; Huyan et al., 2019; Jin et al., 2021). A recent randomized study found that DEX used during a daytime operation can better improve postoperative sleep quality in patients undergoing laparoscopic abdominal surgeries (Song et al., 2019). However, patients undergoing abdominal surgery usually suffer from reduced gastrointestinal transit and prohibition of intestinal peristalsis, which is described as a temporary impairment of gastrointestinal motility (Cho et al., 2017; Turkay et al., 2020). These patients present with a variety of clinical symptoms, including postoperative nausea and vomiting (PONV), delayed passage of flatus and feces, and the inability to tolerate solid food, resulting in an extended recovery time (Yang et al., 2016). In colorectal cancer surgery, changes in anatomical structure may promote intestinal obstruction and have a significant impact on gastrointestinal motility function (Bragg et al., 2015). It was found that α_2_ adrenergic agonists reduce vagally mediated gastric and small bowel motility (Maze and Tranquilli, 1991). However, the effect of DEX on gastrointestinal motility remains controversial (Iirola et al., 2011; Chen et al., 2016).

DEX has been effectively used for patient-controlled analgesia (PCA). Whether DEX has a positive impact on postoperative sleep quality and gastrointestinal motility function in patients after colorectal cancer surgery remains uninvestigated. The purpose of this study was to determine the effects of DEX combined with sufentanil for PCA on postoperative sleep quality and gastrointestinal motility function in patients after colorectal cancer surgery.

## Methods

### Trial design

This study was a single-center, prospective, randomized, double-blinded, controlled trial performed in Harbin Medical University Cancer Hospital from 1 July 2019 to 30 December 2019. The study was approved by the Ethics Committee of Harbin Medical University Cancer Hospital (2019-189-ⅡT) and registered with the Chinese Clinical Trial Registry (ChiCTR2000032601). Written informed consent was obtained from all subjects participating in this trial before surgery.

### Participants

Patients who underwent elective colorectal cancer surgery and were expected to require postoperative PCA were enrolled in this study. The inclusion criteria were an age of over 18 years and an American Society of Anesthesiologists (ASA) physical status of I-III. The exclusion criteria included long-term use of opioids, sedatives, antidepressants, or anxiolytic drugs prior to surgery; drug addiction; a preoperative history of schizophrenia, epilepsy, Parkinsonism, or myasthenia gravis; a preoperative sleep disorder; the use of sleep-promoting medications; sleep apnea syndrome; the inability to provide informed consent (coma, profound dementia, or language barriers); sick sinus syndrome, severe sinus bradycardia (<50/min), or a second-degree or greater atrioventricular block without a pacemaker; serious hepatic dysfunction (Child–Pugh class C); serious renal dysfunction (undergoing dialysis before surgery); and an allergy to opioid analgesics or DEX.

### Randomization and masking

Patients were randomized through the use of a random number table. Enrolled patients were randomly allocated into the DEX 0 μg group, DEX 200 μg group or DEX 400 μg group. To explore the effects of DEX for PCA on gastrointestinal motility function after different surgical categories, patients in the three groups were further divided into laparoscopic surgery and open surgery subgroups. Two investigators carried out this study in a blinded manner. The first investigator was responsible for enrollment and the assignment of participants to groups by randomization. The variables were recorded by the second investigator, who was blinded to each subject’s assigned group. Participants were blinded to group assignments throughout the study period. In case of an emergency (unexpected or rapid deterioration of the patient’s clinical status), physicians were allowed to request the unmasking of the treatment assignment or the adjusting or interrupting of the study, if necessary. In such a case, the patient was excluded from the final analysis.

### Anesthesia and analgesia procedures

Anesthesia methods were standardized in all three groups. General anesthesia was induced with propofol 1.0–1.5 mg/kg, sufentanil 0.3–0.5 μg/kg, and cisatracurium 0.15 mg/kg. Tracheal intubation was facilitated after 3 min of cisatracurium administration and was connected to a ventilator. The ventilation rate was 12 breaths/min. The tidal volume (8–10 ml/kg) was adjusted to maintain the end-tidal CO_2_ (EtCO_2_) level at 35–45 mmHg. Propofol 4–10 mg/kg/h and remifentanil 5–20 μg/kg/h were administered and adjusted according to the hemodynamic responses to maintain a bispectral index (BIS) between 40 and 60 during anesthesia. The neuromuscular blockade was maintained by intermittent injection of 0.05 mg/kg cisatracurium as needed. All patients in the three groups received PCA after surgery. The PCA regimen consisted of sufentanil 150 μg (in 300 ml normal saline), which was set up as a continuous infusion dose of 4 ml/h with a bolus dose of 3 ml (if needed), with a lock-out time of 15 min in all groups. DEX (200 and 400 μg) was mixed with PCA in the DEX 200 μg group and DEX 400 μg group, respectively. The PCA pump was used for up to 3 days after surgery until all of the solution was exhausted. Refilling the PCA pump was not allowed for any group. The acute rescue analgesic drug flurbiprofen axetil (50 mg i.v.), was given when the visual analog scale (VAS: 0, no pain; 10, severe pain) score was more than 4 after three continuous bolus infusions of PCA.

### Outcome measures

During the postoperative period, the following variables were assessed: the Athens Insomnia Scale (AIS) score; the VAS score, both at rest and with movement at 2, 24 and 48 h after surgery; the time of first flatus, first feces and first diet; the dosage of consumed sufentanil; the number of PCA attempts; the rate of rescue analgesia; side effects (PONV, bradycardia or hypotension); and the length of postoperative hospital stay.

The primary outcome of this study was sleep quality for 7 continuous days after surgery. Postoperative sleep quality was assessed with the AIS score and the incidence of postoperative sleep disturbance. The AIS is a self-reported questionnaire that quantifies sleep disturbances in accordance with the criteria set by the International Classification of Diseases (ICD-10). It consists of 8 items: sleep induction, awakenings during the night, early morning awakening, total sleep time, overall quality of sleep, sleep quality well-being, functioning capacity, and daytime sleepiness. Item scores range from 0 (no problem at all) to 3 (very serious problem), for a total score of 0–24. The AIS scores were recorded at 5:00 p.m. the following day by investigator who was blinded to each subject’s assigned group. A lower AIS score indicates better sleep quality. A total score of > 6 points reflects the diagnosis of sleep disturbance. The overall incidence of postoperative sleep disturbance was defined as the proportion of score > 6 points within 7 days after surgery.

The secondary outcome was postoperative gastrointestinal motility recovery based on the time of first flatus, first feces and first diet. Other outcomes included postoperative pain intensity at rest and with movement at 2, 24 and 48 h after surgery; the dosage of consumed sufentanil of PCA; the number of PCA attempts at 24 and 48 h after surgery and the dosage of acute rescue analgesic drugs (flurbiprofen axetil); side effects (PONV, bradycardia or hypotension) and postoperative hospital stay. Hypotension was defined as a mean arterial pressure of 30% below baseline, and bradycardia was defined as a heart rate of < 50 beats/min. The clinical characteristics of patients, such as age, sex, height, body weight, surgical site (rectal vs. colon), and surgical category (laparoscopic vs. open) were recorded.

### Statistical analysis

Based on an expected incidence of the primary endpoint as a 16.6% occurrence of postoperative sleep disturbance in the DEX 0 μg group, 7.1% in the DEX 200 μg group and 4.8% in the DEX 400 μg group in our preliminary experiments, the minimum sample size was calculated. For a two-sided difference with 80% power at the 0.05 significance level, sixty-one participants in each group were required. Considering subjects who may be lost to follow-up or may otherwise drop out, seventy participants in each group were enrolled.

IBM SPSS Statistics version 26.0 software (IBM Corp., Armonk, NY, United States) was used to perform statistical analyses. The Kolmogorov–Smirnov test was applied to assess the distribution of the variables. Homogeneity of variance was compared among the three groups by Levene tests. Continuous variables are presented as the mean ± SD. Stratified continous variables are presented as the median with an interquartile range. Categorical variables are presented as a percentage. The incidence of sleep disturbance, rate of rescue analgesia, sex, ASA, surgical site, surgical category, and side effects among groups were analyzed using the chi-squared test or Fisher’s exact test. VAS score and PCA attempts were analyzed using a Kruskal–Wallis H test. The surgery time, AIS score, time to postoperative first flatus, time to postoperative first feces, time to postoperative first diet, dosage of consumed sufentanil, and general characteristics of the patients, including age, height, and weight, were analyzed using a one-way ANOVA followed by a *post hoc* least significant differences test. Time to postoperative first flatus, first feces, and first diet classified by surgical category were analyzed using a two-way ANOVA. *p* < 0.05 was considered statistically significant.

## Results

### Patient characteristics

**FIGURE 1:**
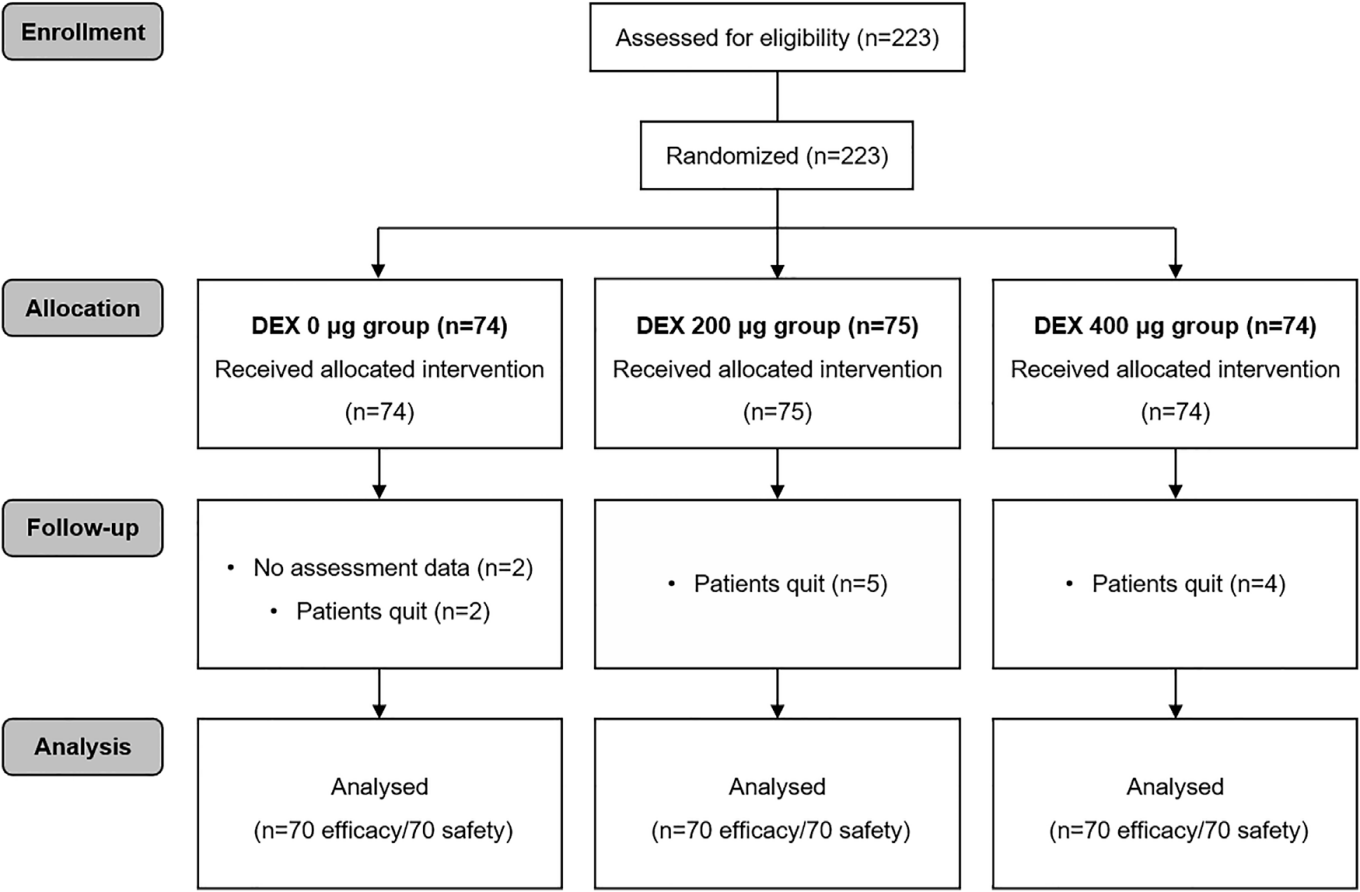
CONSORT diagram. DEX, dexmedetomidine.

During the study period, 223 patients were involved (Figure 1). Thirteen patients were subsequently excluded from the analysis. Eleven of these patients voluntarily discontinued the trial, and the assessment data for 2 patients were unavailable. The study included 210 cases eligible for analysis, with 70 cases in each group. There were no differences in the clinical characteristics of the patients among the three groups, including age, sex, height, body weight, ASA physical status, surgical site surgical category and surgery time (Table 1).

**TABLE 1:** Clinical characteristics of patients in the three groups.

	DEX 0 μg (*n* = 70)	DEX 200 μg (*n* = 70)	DEX 400 μg (*n* = 70)	*p* value
Age, y	60.0 ± 8.9	58.8 ± 10.2	60.2 ± 8.1	0.620
Sex, M/F	38/32	49/21	49/21	0.080
Height, cm	166.0 ± 8.1	167.2 ± 7.0	168.0 ± 7.0	0.278
Body weight, kg	66.2 ± 11.0	66.8 ± 9.7	69.8 ± 11.9	0.106
ASA, n (%)				0.775
I	0 (−)	1 (1.4%)	0 (−)	
II	68 (97.1%)	69 (98.6%)	69 (98.6%)	
III	2 (2.9%)	0 (−)	1 (1.4%)	
Surgical site, n (%)				0.237
Rectal	38 (54.3%)	33 (47.1%)	43 (61.4%)	
Colon	32 (45.7%)	37 (52.9%)	27 (38.6%)	
Surgical category, n (%)				0.494
Laparoscopic	40 (57.1%)	36 (51.4%)	33 (47.1%)	
Open	30 (42.9%)	34 (48.6%)	37 (52.9%)	
Surgery time, min				0.988
Laparoscopic	151.3 ± 48.4	152.5 ± 45.2	137.9 ± 35.3	
Open	119.3 ± 35.8	119.4 ± 38.1	130.0 ± 54.2	

DEX, dexmedetomidine; ASA, american society of anesthesiologist.

### The primary outcome

**FIGURE 2:**
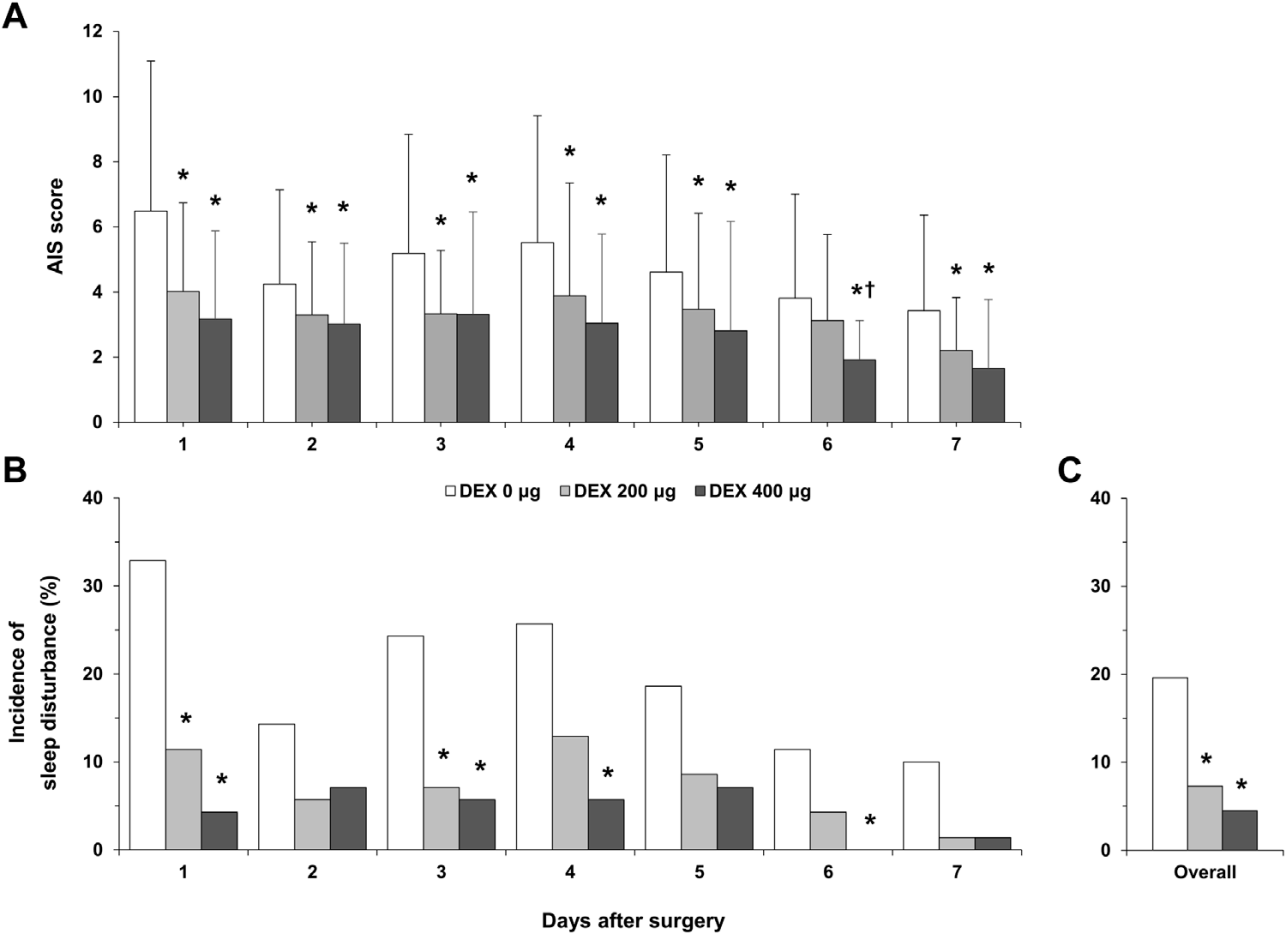
Postoperative sleep quality. **(A)**, AIS score over 7 postoperative days; **(B)**, incidence of postoperative sleep disturbance over 7 postoperative days; **(C)**, overall incidence of postoperative sleep disturbance during the first 7 days after surgery. DEX, dexmedetomidine; AIS, Athens Insomnia Scale; *p < 0.05, vs. DEX 0 μg; ^†^ *p* < 0.05, vs. DEX 200 μg.

Compared with that of the DEX 0 μg group, the AIS score in the DEX 200 μg group was significantly lower on postoperative days 1, 2, 3, 4, 5, and 7 (*p* < 0.05, Figure 2A). The AIS score in the DEX 400 μg group was significantly lower than that in the DEX 0 μg group within all 7 days after surgery (*p* < 0.05). The AIS score in the DEX 400 μg group was significantly lower than that in the DEX 200 μg group on postoperative day 6 (*p* = 0.004). The incidence of postoperative sleep disturbance in the DEX 200 μg group was significantly lower than that in the DEX 0 μg group 1 and 3 days after surgery (*p* < 0.05, Figure 2B). The incidence of postoperative sleep disturbance in the DEX 400 μg group was significantly lower than that in the DEX 0 μg group 1, 3, 4, and 6 days after surgery (*p* < 0.05, Figure 2B). There were no differences in the incidence of postoperative sleep disturbance between the DEX 200 µg group and DEX 400 µg group. The overall incidence of postoperative sleep disturbance during the first 7 days after surgery was significantly lower in the DEX 200 µg and DEX 400 µg groups than in the DEX 0 µg group (7.3%, 4.5% vs. 19.6%, *p* < 0.001, Figure 2C).

### The secondary outcomes

There were no significant differences in postoperative gastrointestinal motility among the three groups in the total surgical category (Table 2). In the laparoscopic surgery group, the time of postoperative first flatus and first feces was significantly longer in the DEX 400 μg group than in the DEX 0 μg group. There was no significant difference in the time of the postoperative first diet among all groups.

**TABLE 2:** Postoperative gastrointestinal motility recovery.

	DEX 0 μg (*n* = 70)	DEX 200 μg (*n* = 70)	DEX 400 μg (*n* = 70)	*p* value
First flatus, h				
Total	61.2 ± 29.5	64.8 ± 29.0	74.8 ± 47.7	0.075
Laparoscopic	58.3 ± 5.8	64.8 ± 6.1	78.4 ± 6.4*	0.044
Open	65.0 ± 6.7	64.9 ± 6.3	71.6 ± 6.0	0.683
First feces, h				
Total	82.9 ± 35.6	87.0 ± 35.4	97.6 ± 49.0	0.088
Laparoscopic	76.5 ± 6.4	87.8 ± 6.8	102.5 ± 7.1*	0.049
Open	91.5 ± 7.4	86.3 ± 7.0	93.4 ± 6.7	0.749
First diet, h				
Total	107.3 ± 34.2	101.0 ± 37.4	115.3 ± 53.0	0.135
Laparoscopic	107.2 ± 6.7	98.4 ± 7.1	121.2 ± 7.4	0.153
Open	107.5 ± 7.8	103.7 ± 7.3	110.2 ± 7.0	0.814

DEX, dexmedetomidine. *p < 0.05, vs. DEX 0 µg.

The VAS score at rest or with movement at 2, 24 and 48 h after surgery showed no significant differences among the three groups (Table 3). There was no difference in the dosage of consumed sufentanil among all groups 24 and 48 h after surgery. The number of PCA attempts in the DEX 200 μg group was significantly lower than that in the DEX 0 μg and DEX 400 μg groups at 24 h (*p* = 0.013) and 48 h (*p* = 0.009) after surgery. There were no significant differences in the rate of rescue analgesia or postoperative hospital stay among the groups. No episodes of PONV, bradycardia or hypotension were recorded.

**TABLE 3:** Other outcomes of patients in the three groups.

	DEX 0 μg (*n* = 70)	DEX 200 μg (*n* = 70)	DEX 400 μg (*n* = 70)	*p* value
VAS score at rest				
2 h after surgery	0 (0.5)	0 (0.2)	0 (0.2)	0.727
24 h after surgery	1 (0.4)	1 (0.3)	1 (0.4)	0.960
48 h after surgery	1 (0.5)	0 (0.2)	1 (0.4)	0.067
VAS score with movement				
2 h after surgery	0 (0.5)	1 (0.5)	0 (0.3)	0.260
24 h after surgery	5 (5.5)	5 (5.5)	5 (5.5)	0.260
48 h after surgery	5 (5.5)	5 (5.5)	5 (5.5)	0.591
PCA pump				
24 h after surgery				
Sufentanil, μg	57.3 ± 7.8	54.5 ± 5.8	53.9 ± 6.3	0.202
PCA attempts, n	6.9 ± 7.2	3.9 ± 4.6*	5.4 ± 5.9	0.013
48 h after surgery				
Sufentanil, μg	99.6 ± 19.8	93.1 ± 16.9	95.6 ± 15.7	0.089
PCA attempts, n	10.9 ± 11.0	6.3 ± 6.0*	8.9 ± 8.4	0.009
Rate of rescue analgesia, n (%)	7 (10.0%)	3 (4.3%)	3 (4.3%)	0.269
PONV, n (%)	7 (10.0%)	6 (8.6%)	1 (1.4%)	0.093
Postoperative hospital stay, d	9.9 ± 4.4	10.3 ± 5.4	9.1 ± 2.1	0.231

DEX, dexmedetomidine; VAS, visual analog scale; PCA, patient-controlled analgesia; PONV, postoperative nausea and vomiting. *p < 0.05, vs. DEX 0 μg.

## Discussion

This randomized clinical trial investigated the effect of 0, 200, and 400 μg of DEX combined with sufentanil for PCA on postoperative sleep quality and the recovery of gastrointestinal motility function in patients after colorectal cancer surgery. Our results demonstrated that the continuous infusion of 200 and 400 μg of DEX for PCA after surgery improves sleep quality in the first 7 days. The recovery of postoperative gastrointestinal motility function was not significantly different across the whole sample (both laparoscopic and open surgeries included) among the three comparison groups. However, in patients who underwent laparoscopic surgery, the DEX 400 μg group inhibited postoperative recovery of gastrointestinal motility more than the DEX 0 μg group. Postoperative pain intensity, side effects and postoperative hospital stay did not differ among the three groups. According to this study, DEX 200 μg with sufentanil for PCA is preferred in patients with colorectal cancer surgery for improving postoperative sleep quality and provides effective analgesia without affecting gastrointestinal motility function.

Studies have shown that DEX used for PCA at a rate of 0.03 μg/kg/h improves postoperative sleep quality (Chen et al., 2017; Hong et al., 2021). The total dosage of DEX used in this study was 0.03–0.05 and 0.07–0.09 μg/kg/h according to the body weight of enrolled patients in the DEX 200 μg group and DEX 400 μg group, respectively, which was higher than that used in the above report. The higher dosage of DEX used for PCA in this study was due to the results of our previous study, where 200 and 400 μg of DEX for PCA reduced the incidence of postoperative delirium in elderly patients without increasing side effects, and 100 μg of DEX (0.02–0.03 μg/kg/h) had no effect on the improvement of postoperative delirium (Zhao et al., 2020).

Sleep is an active, complex process that is necessary for mental and physical restoration. Sleep disturbances are known to result in poor healing, reduced cognitive function, and an increased chance of cancer recurrence (Weinhouse et al., 2009; Otte et al., 2015). One study demonstrated that postoperative sleep disturbance is most serious within the first 3 days after surgery, which manifests with decreased sleep time, increased awakenings, and disturbance of sleep rhythm (Knill et al., 1990). Patients frequently report postoperative sleep disturbance in response to surgical stress; 42% of patients complained of unsatisfactory sleep after orthopedic, vascular, and general surgery (vs. 28% the night before surgery), and their sleep remained unsatisfactory after 4 days in 23% of cases (Chouchou et al., 2014). In our study, the overall incidence of postoperative sleep disturbance within 7 days after surgery was 19.6%, and both 200 and 400 μg of DEX used for PCA reduced the incidence of sleep disturbances (7.3% vs. 4.5%), improving the sleep quality of patients. Postoperative sleep disturbances may be the result of a variety of factors, including pain and hospital environment-related factors, noise and light exposure from procedures or intensive monitoring during the night (Dolan et al., 2016). Postoperative pain is one of the most common factors affecting postoperative sleep quality (Miller et al., 2015; Dolan et al., 2016; Chen et al., 2017). However, in the present study, postoperative analgesia was similar among the groups. Thus, the improvement of postoperative sleep quality by DEX was not due to analgesia. The locus coeruleus nucleus is the site that receives external stimuli and sleep arousal (Song et al., 2017). DEX exerts its hypnotic action through selective activation of central pre- and postsynaptic adrenergic receptors in the locus coeruleus (Bamgbade, 2006). DEX inhibits locus coeruleus-derived noradrenergic neurotransmission to the ventrolateral preoptic nucleus, thus disinhibiting the ventrolateral preoptic nucleus and provoking an inhibition of cortical arousal nuclei (Guldenmund et al., 2017).

Previous studies have shown that the intraoperative use of DEX was associated with a shorter gastrointestinal motility recovery time than the use of a placebo (Chen et al., 2016; Mah et al., 2021). It was found that the intraoperative use of DEX was associated with reductions in time of first flatus, first feces, and the return to a regular solid diet in laparoscopic resection of colorectal cancer (Chen et al., 2016). A recent multicenter, placebo-controlled randomized clinical trial also suggested that the administration of intraoperative DEX reduced the time of first flatus and first feces in patients undergoing open and laparoscopic abdominal surgeries (Mah et al., 2021). However, the reported effects of DEX on gastrointestinal motility function are inconsistent. In patients undergoing abdominal hysterectomy, no difference in time to first bowel sounds and flatus was observed after receipt of intraoperative DEX (Xu et al., 2017). Two preclinical studies and one study involving healthy participants found that DEX inhibits gastrointestinal motility function (Asai et al., 1998; Herbert et al., 2002; Iirola et al., 2011). DEX markedly inhibited gastric emptying and gastrointestinal transit when healthy participants received 1 μg/kg of DEX over 20 min followed by a continuous infusion of 0.7 μg/kg/h for 190 min (Iirola et al., 2011). The current study found that, compared with the DEX 0 μg group, the use of 400 μg of DEX combined with sufentanil for PCA prolonged the time to the first flatus (74.8 ± 47.7 vs. 61.2 ± 29.5), first feces (97.6 ± 49.0 vs. 82.9 ± 35.6) and first diet (115.3 ± 53.0 vs. 107.3 ± 34.2), although there were no statistically significant differences in the recovery time of gastrointestinal motility function in either the laparoscopic or open surgical categories. However, based on the subgroup analysis, the effect of DEX on gastrointestinal motility function was dependent on the surgical category. In the laparoscopic groups, the time of postoperative first flatus and feces of the DEX 400 μg group was longer than that in the DEX 0 μg group, and there was no difference between the DEX 200 μg and DEX 0 μg groups, indicating that 400 μg of DEX, but not 200 μg of DEX, inhibited the recovery of postoperative gastrointestinal function. In the open surgery groups, the recovery of gastrointestinal motility function was not different between DEX groups.

The effects of DEX on postoperative gastrointestinal function may be related to the dosage and the time of administration. A preclinical study revealed that DEX concentration dependently inhibited peristalsis of the guinea pig ileum *in vitro*, and that the inhibition was caused by the interaction with α_2_-adrenoceptors (Herbert et al., 2002). Two clinical studies with opposite results demonstrated the dosage-dependent effect of DEX. Patients who received DEX at a total dosage of 3 μg/kg showed inhibited gastric emptying and gastrointestinal transit (Iirola et al., 2011). However, gastric emptying was not delayed when the total dosage was 1 μg/kg (Memiş et al., 2006). Low-dose DEX may improve gastrointestinal transit by acting on central α_2_-adrenoceptor agonists to reduce sympathetic tone (Cho et al., 2015). High-dose DEX may inhibit peristalsis by activating inhibitory α_1_-adrenoceptors located postsynaptically on the smooth muscle or by activating inhibitory α_2_-adrenoceptors on excitatory cholinergic pathways in the enteric nervous system, such as opioid, purinergic, and nitrergic neurons (De Ponti et al., 1996). The effects of DEX on postoperative gastrointestinal function were different depending on the time of administration. Low perfusion of intestinal smooth muscle disturbed intestinal motility during surgery. DEX improved postoperative gastrointestinal function by its global hemodynamic stabilizing effect, preventing the violent alteration of gastrointestinal microcirculation, attenuating intestinal ischemia–reperfusion injury, and improving stress response (Kiliç et al., 2012; Zhang et al., 2012). However, during the nonsurgical period, DEX inhibits gastrointestinal function by affecting the α_2_-adrenoceptor of enteric neurons (Herbert et al., 2002; Iirola et al., 2011). Furthermore, the effects of DEX may vary depending on the degree of physiological impairment in the participant. Abdominal open surgery causes severe hemodynamic changes and extensive trauma, which is a representative of long-term major surgery. Evidence suggests that compared to laparoscopic surgery, open approaches quickly induce an inflammatory cascade and stress responses by causing greater tissue injury (Raygor et al., 2015). Under physiological conditions, DEX inhibited gastrointestinal motility in the study of healthy participants (Iirola et al., 2011). In a study involving critically ill patients, no difference in gastric emptying time was observed after receipt of DEX vs. propofol (Memiş et al., 2006). In the present study, high-dose DEX did inhibit the recovery time of postoperative first flatus and first feces in laparoscopic surgery rather than open surgery, suggesting the dose and the degree of physiological impairment are relevant in determining the benefits of DEX associated with return of gastrointestinal function.

DEX at a rate of 0.02–0.05 μg/kg/h with sufentanil at a rate of 0.015–0.02 μg/kg/h used for PCA potentiated the analgesic effect of sufentanil and reduced the sufentanil consumption in abdominal surgery (Feng et al., 2019). In the present study, the VAS scores and sufentanil consumption was not statistically different among three groups, which is consistent with our previous study (Zhao et al., 2020). The dosage of sufentanil used in this study was 0.03 μg/kg/h (total dosage up to 150 μg) according to the body weight of enrolled patients, which was higher than that used in the above report (Feng et al., 2019). This indicated that dosage of sufentanil in our study for PCA was sufficient for postoperative analgesia, the combination of DEX did not further potentiate the analgesia effect of sufentanil. A study with a higher dosage of sufentanil (a rate of 0.04 μg/kg/h) supports the result that DEX did not potentiate sufentanil in PCA (Chen et al., 2016). The PCA was set up at a 3 ml bolus if needed with a background infusion dosage of 4 ml/h for up to 3 days. Although the PCA attempts varied among groups, the relatively smaller proportion of bolus consumption compared with the consistent background infusion of the three groups did not result in a statistically difference in sufentanil consumption among three groups. The number of additional rescue analgesia requirement in the DEX 0 μg group was higher than the DEX 200 μg group and the DEX 400 μg group, but it was no statistics difference. The number of PCA attempts in the DEX 200 μg group were significantly lower than in the 0 μg group. There were no statistics difference between the DEX 200 μg group and DEX 400 μg group or between the DEX 0 μg group and the 400 μg group. The DEX 200 μg group had less number of the PCA attempts while with large standard deviation.

This present study had a couple of limitations. First, there was no long-term (>7 days postoperatively) evaluation of postoperative sleep quality. A study showed that 25% of patients had not returned to normal sleep quality 2 weeks after discharge (Chouchou et al., 2014). Considering the short hospital stays of the patients, this analysis was limited to evaluating sleep quality within 7 days after surgery. Second, the sample size estimation in our study was based on postoperative sleep disturbance, which was powered solely as the primary endpoint in our present study. It is worth noting that the 400 μg group prolonged the time to the first flatus, first feces and first diet for almost 10 h in total surgical categories, although the differences were only numerically different. It cannot be excluded that statistically significant differences in secondary outcomes may have become apparent after the inclusion of a larger sample size.

## Conclusion

Our study indicated that, compared with the use of sufentanil alone for PCA, a continuous infusion of DEX (200 or 400 μg) with sufentanil for PCA for up to 3 days significantly improved sleep quality in the first 7 days for patients after colorectal cancer surgery without increasing any side effects or prolonging their hospital stay. Compared with 400 μg of DEX, 200 μg of DEX was better at improving postoperative sleep quality without affecting gastrointestinal motility function in patients who underwent laparoscopic surgery. According to this study, 200 μg of DEX with sufentanil for PCA is preferred in patients with colorectal cancer surgery for improving postoperative sleep quality and providing effective analgesia without affecting gastrointestinal motility function.

## Data Availability

The raw data supporting the conclusion of this article will be made available by the authors, without undue reservation.
